# A New Class of Smart Gadolinium Contrast Agent for Tissue pH Probing Using Magnetic Resonance Imaging

**DOI:** 10.3390/molecules25071513

**Published:** 2020-03-26

**Authors:** Fouzi Mouffouk, Hacene Serrai, Sourav Bhaduri, Rik Achten, Mozhdeh Seyyedhamzeh, Ali A. Husain, Abdullah Alhendal, Mohammed Zourob

**Affiliations:** 1 Department of Chemistry, Kuwait University, P.O. Box 5969, Safat 13060, Kuwait; m.hamzeh@ku.edu.kw (M.S.); ali.husain@ku.edu.kw (A.A.H.); abdullah.alhendal@ku.edu.kw (A.A.); 2 Department of Diagnostic Sciences, University of Ghent, 9000 Gent, Belgium; Hacene.Serrai@ugent.be (H.S.); sourav.bhaduri@ugent.be (S.B.); Rik.Achten@ugent.be (R.A.); 3 Department of Chemistry, Alfaisal University, Riyadh 12713, Saudi Arabia; 4 King Faisal Specialist Hospital and Research Center, Riyadh 12713, Saudi Arabia

**Keywords:** cancer imaging, MRI, pH, sensors

## Abstract

Detecting tissue pH in vivo is extremely vital for medical diagnosis and formulation of treatment decisions. To this end, many investigations have been carried out to develop an accurate and efficient method of in vivo pH measurement. Most of the techniques developed so far suffer from inadequate accuracy, due to poor sensitivity at low concentration of the target or nonspecific interactions within the tissue matrix. To overcome these issues, we describe herein the development of a simple, yet reliable, way to estimate pH with high precision using a Gd(III)-DOTA-silyl-based acid-labile group as a pH-sensitive contrast agent with Magnetic Resonance Imaging (MRI). With this method, a change in $T_{1}$ weighted image intensity of the newly developed pH-sensitive contrast is directly linked to the proton concentration in the media. As a result, we were able estimate the pH of the target with 95% reliability.

## 1. Introduction

Numerous physiological disorders, such as renal failure, hepatic problems, heart trauma, chronic obstructive pulmonary disease and cancer cell metastases are all directly associated with tissue acidity (pH). The need for accurate techniques capable of detecting pH has therefore triggered a wide range of research efforts [1,2]. Several methods of pH measurement with modalities other than the MRI technique have been investigated comprehensively.

For example, optical imaging with a fluorescence probe has been used to measure the pH by measuring the ratio of fluorescence signals at different fluorescence lifetimes [3], or at different emission wavelengths [4]. Unfortunately, due to the light incursion depth limitation, optical imaging cannot assess the pH of cavernous tissues, which severely restricts the efficiency of this imaging modality to solitary surface-manageable cancer tissue [5]. Bioconjugation of the radioactive nuclide (^64^Cu) to pH-sensitive peptides (pHLIP) has been examined as a contrast agent for Positron Emission Tomography (PET) to assess tissue pH [6]. However, the pH values obtained using this modality lack accuracy [7]. Electron paramagnetic resonance (EPR) spectroscopy is another method that has been investigated as a potential candidate for tissue pH measurement [8]. Here, the tissue has to be irradiated with high energy to conduct EPR studies, which limits this modality to small animal models and human tissue samples.

Chemical Exchange Saturation Transfer (CEST) is a contrast enhancement technique that enables the indirect detection of molecules with exchangeable protons and exchange-related properties [9,10,11]. This technique helps to detect concentrations of metabolites, based on magnetization exchange between exchangeable protons [12]. Endogenous metabolites with exchangeable protons, including proteins with amide protons, glycosaminoglycans (GAG), glycogen, myo-inositol (MI), glutamate (Glu), creatine (Cr) and other markers, have been identified as potential in vivo endogenous CEST agents [13]. The direct effect of pH on chemical exchange rates makes CEST imaging a good technique to assess change in pH in vivo. As a result, CEST imaging has been used to quantify changes in pH [14]. Nonetheless, CEST-based pH quantification has certain limitations. In an in vitro setting, several algorithms have been developed to accurately measure and quantify differences in pH [15]. The problem is, CEST contrast depends on several parameters including labile proton concentration, temperature, water content, the spin-lattice relaxation ($T_{1}$) of water, and saturation parameters which affect the chemical environment of the exchanging protons. This makes in vivo pH quantification a significantly more challenging method because it necessitates these factors be taken into account. Similarly, certain MRI contrast agents are pH-sensitive, due to the ability of metal chelates to hinder water accessibility to metal ions (such as the Gd ion) at a low pH level. This mode of pH measurement requires a second contrast with no pH dependence, in order to convert the $T_{1}$ weighted MRI signal into pH values [16]. For example, pH responsive contrast (Gd(DOTA)-4AmP5-) and pH unresponsive contrast (Gd(DOTP)5-) were both used to determine the pH within the range of pH 6.0 to 8.5. [14]. The basis of this technique is the assumption that the measured temporally dynamic concentration of Gd(DOTP)5- is similar to Gd-DOTA-4AmP. As a result, it allows for the determination of the $T_{1}$ weighted MRI signal of Gd-DOTA-4AmP which will be used to determine the pH of the tissue. However, the time lapse between the injections of the two contrasts is problematic since their bio-distribution is not the same, which imposes a question about the validity of the assumption that both agents have identical temporally dynamic concentration [15]. This makes it a questionable technique.

The work presented in this paper describes the determination of endogenous pH, using only Gd^+3^ concentration of a single contrast measured by MRI. The Gd(III)-DOTA-silyl complex has a low signal intensity at neutral pH due to the shielding effect of the silyl groups. However, at lower pH, the silyl protecting groups get cleaved, leaving the DOTA-tetra(amides) complex exposed to interact with water molecules, leading to a signal intensity increase on *T1*-weighted MR images (signal decay decrease), where the intensity of Gd^+3^ is directly linked to proton concentration, which eventually enables an accurate estimation of the pH.

## 2. Results and Discussion

**Scheme 1:**
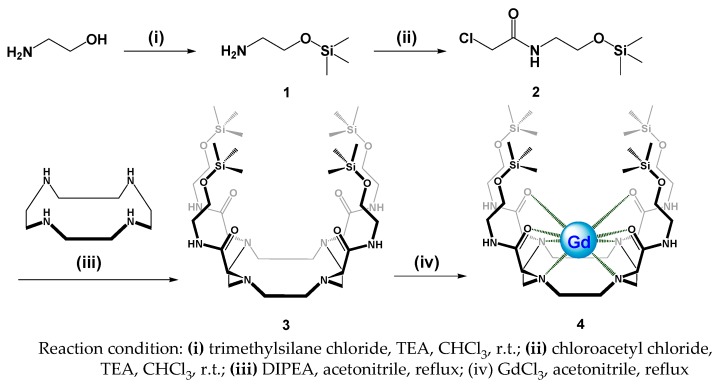
Synthesis of Gd(III)-DOTA-silyl complex (**4**).

The focus point of this approach can be summarized in the following points: first the establishment of an acidic labile shield, i.e., trimethylsilyl ether (TMS), containing amide substituents suitable to form a stable complex with Gd^+3^ cation through the ligating groups. Then the use of the Equation (1) to convert $T_{1}$ weighted MRI signal into Gd concentration and finally the conversion of these concentrations into pH values using Equation (3). All Precursors fabrication steps of the pH sensitive contrast agent are carried out using classic organic synthesis protocols as illustrated in Scheme 1. The presence of the multiple bulky alkylated silane groups and their spatial directionality provided by the cyclen skeleton, creates umbrella-like structure (complex **4**) capable of shielding the Gd ion within the cyclen cavity from the outer environment, especially water molecules.

The trimethylsilyl TMS group is used for protection of all kinds of functional groups, and even today remains pre-eminent. Because of this sensitivity, cleavage of TMS derivatives can be achieved easily by basic or acidic hydrolysis, or via solvolysis.

**Figure 1:**
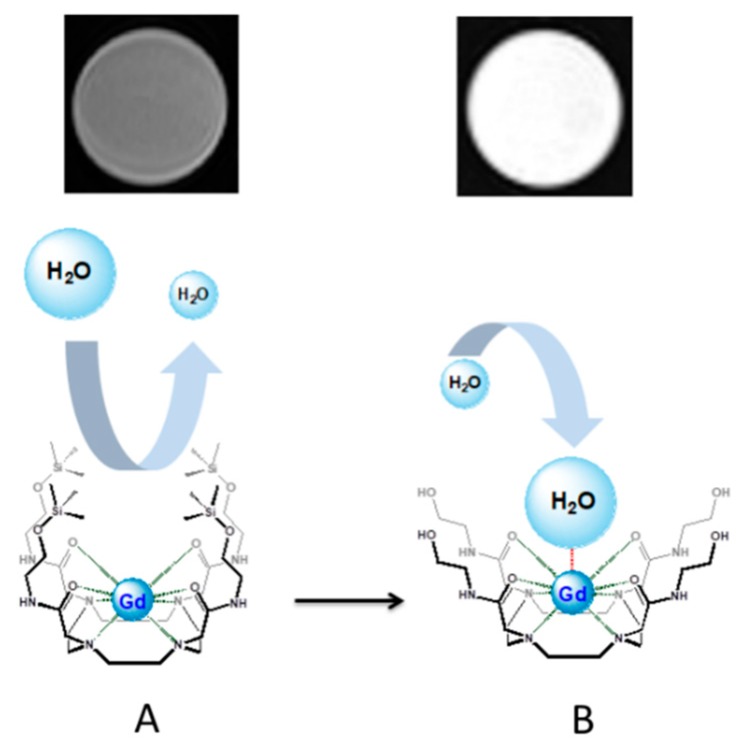
*T_1_* weighted in vitro of Gd(III)-DOTA-silyl complex (**A**) at neutral condition (**B**) at acidic condition.

The key function of this method relies on the ability of a silyl protecting group to prevent water molecules from accessing the Gd^+3^ ions by forming a hydrophobic barrier around the Gd^+3^ complex “off stat”. However, at slightly low pH-values (pH < 7), these groups get cleaved rapidly, allowing the water molecules to interact with the Gd^+3^ ion, which provokes a change in the magnetic properties of the water protons surrounding this complex “on stat”. The rate of hydrolysis of the silyl group depends heavily on both electronic and steric effects. It is well documented that the stability of trimethylsilyl ether (Me_3_SiOR) to acidic hydrolysis increases with the increase in the electron-withdrawing nature of R. Since the ether moiety of the contrast (DOTA-tetraamide) is highly electron-withdrawing, it renders these contrasts more stable in acidic condition compare to classic TMS ether (alcohol). Also, the increase in the steric size and hydrophobicity of the alcoholic part of the Gd^3+^complex will decrease the rate of solvolysis of these groups by water, which extends their stability in aqueous solution for more than 2 h. The obtained MRI image demonstrates clearly this stability in aqueous solution (Figure 1A), considering that this image has been taken 2 h after the incubation in aqueous containing solutions. Conversely, in the presence of protons the cleavage take place in les then 30 min as shown in NMR experiment, which leads to a bright image (Figure 1B).

Since the paramagnetic properties of Gadolinium has effects on both longitudinal and transverse relaxation times, the result is shortening of both the $T_{1}$ and $T_{2}$ of tissues in which it accumulates. The parameter $T_{1}$ is the spin–lattice relaxation time representing the time constant for regrowth of longitudinal magnetization, and the parameter $T_{2}$ is the spin–spin relaxation time representing the time constant for dephasing of transverse magnetization. The contrast and brightness of the MRI images are determined by the $T_{1}$ and $T_{2}$ properties of the sample. Contrast agents containing Gd induce both $T_{1}$ and $T_{2}$ relaxation in tissues where they accumulate as a result of dipolar interactions between water nuclei (in tissue) and electron spins in the metal. This effect is called paramagnetic relaxation enhancement. The addition of Gd increases both $R1=\frac{1}{\;T_{1}}$ and $R2=\frac{1}{T_{2}}$ relaxation rates [17,18]. This results in a signal intensity decrease for $T_{1}$ weighted images according to the following Equation (1) [19,20], where S is the signal intensity of image and $S_{0}$ is the initial signal intensity.

(1) $$S=S_{0}\times \left(1-e^{-\frac{TR}{T_{1}}}\right)\times e^{-\frac{TE}{T_{2}}}$$

Linearity of Signal intensity with respect to gadolinium Gd concentration over a clinically relevant range has been observed in prior literature [17,18]. The relative signal intensity method is used, which assumes that the change in concentration (C) is proportional to change in signal intensity (S), as given by Equation (2) [18]:

(2) C=k. S

Therefore, the concentration of the gadolinium in a particular tube can be calculated using the Equation (3), where the proportionality coefficient *k* depends on the pre-contrast relaxation time and remains constant and S is the change in $T_{1}$ weighted image intensity of the particular tube relative to the intensity value of the reference sample.

Gd^+3^ signal intensity is related to the number of silyl groups cleaved from the complex, which also linked directly to the proton concentration present in the vicinity, where each mol of silyl group cleaved corresponds to 1 mol of protons present in the proximity. Therefore, by converting the signal intensity of Gd contrast into concentration using Equation (3), a direct determination of the proton concentration is enabled. Since only a certain number of cleaved silyl group leads to the recovery of $T_{1}$ signal intensity, by multiplying the Gd concentration by the number of cleaved silyl groups we obtain the proton concentration ([H^+^] = n[Gd^+3^]). The pH is then calculated using the formula $pH=-log\left(\left[H^{+}\right]\right)$ where proton concentration [H^+^] is replaced by n[Gd^+3^] to yield Equation (3).

(3) $$pH=-log\left(n\left[Gd^{+3}\right]\right)$$

To assess the ability of Gd(III)-DOTA-silyl to respond to any change in pH condition, two tubes with different pH (4 and 7) were prepared. This was followed by the addition of equal amounts of Gd(III)-DOTA-silyl. In acidic pH (pH = 4.0), the longitudinal relaxation time $T_{1}$ and longitudinal relaxation rate ($R1=\frac{1}{\;T_{1}}$) at a magnetic field strength of 3 Tesla were found to be (131.4 msec, 7.6 × 10^−3^ msec^−1^). However, at pH = 7.0, with the same parameters the readings were different (365 msec, 2.7 × 10^−3^ msec^−1^). The dissimilarity in both readings indicates clearly the sensitivity of the newly developed contrast agent toward the variation in pH condition (Figure 1).

**Figure 2:**
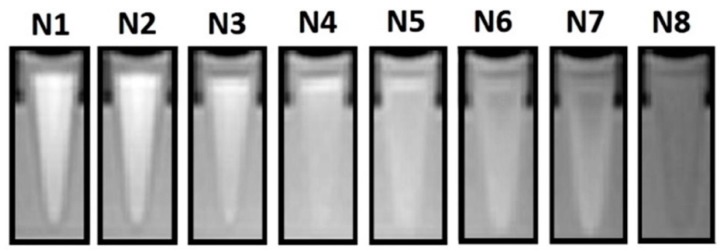
In vitro *T_1_*-weighted MR Images of Gd(III)-DOTA-silyl complex solutions at different pH, or lactic acid concentrations.

To demonstrate the ability of this pH sensitive contrast to estimate the pH value, 8 solutions of different lactic acid concentration were prepared in water with the following pH values (6.5, 6.6, 6.7, 6.8, 6.85, 6.9, 6.95 and 7). Then, 300 µL of Gd(III)-DOTA-silyl complex (5 × 10^−5^ mol/L) in DMSO were added to each tube to form a solution of 1 mL. Thirty minutes after mixing, $T_{1}$ weighted axial images of the solutions were obtained using TR/TE = 250/2.46 ms; matrix size with 256 × 256 resolution (Figure 2).

The obtained data from the $T_{1}$ weighted image intensities were converted into Gd concentration values using a predetermined reference sample (Gd^+3^ = 7.6 × 10^−8^ mol/L) and Equation (2).

The Gd concentration values in different tubes, as measured from the MRI experiment, are given in Table 1. Tube N1, having the highest signal amplitude, gave the highest value of gadolinium concentration, which corresponds to the highest concentration of protons. Tube N8, having the lowest signal amplitude, gave the lowest value of gadolinium concentration, which corresponds to the lowest proton concentration (Table 1).

In order to estimate the pH for each of the 8 tubes, the minimum number of cleaved silyl groups needed for the recovery of the $T_{1}$ weighted signal must be determined. Toward this end, the pH of each tube was assessed based on the assumption that 1, 2 or 3 silyl groups must be removed from the contrast complex in order to increase its signal intensity, which accordingly corresponds to either the 1, 2 or 3 protons required to cleave either 1, 2 or 3 silyl group. This can be interpreted as the concentration of protons [H^+^]. Therefore, to determine the pH, the concentration of the Gd^+3^ obtained by MRI will be multiplied by the number of silyl groups that has been cleaved (1, 2 or 3), which can be translated into a pH-value using Equation (1). The obtained pH values for each tube and assumption are summarized in Table 2.

Thus, by way of comparison of each MRI measured pH with the actual one (measured theoretically and by a pH meter), we observed that in the cases of 1 and 2 protons assumption (multiplication of the Gd^+3^ concentration by 1 or 2), the pH of all tubes did not match the actual pH, which means cleaving 1 or 2 silyl groups is not enough to fully recover the $T_{1}$-weighted signal of the complex. A statistical test (*t-test*) was conducted to compare the means of MRI measured pH values to the actual pH values. The t-test confirmed that the cases of 1 and 2 protons are dissimilar to the actual pH (null hypothesis rejected for t-values above 2.145, degree of freedom 14). However, in the case of multiplying the Gd^+3^ concentration by 3, the obtained pH values were statistically similar to the actual pH values with 95% reliability (t-values 1.54), This implies that cleaving 3 silyl groups is the least number that would be required to recover the $T_{1}$-weighted signal from the complex.

To confirm our assumption, three separate samples were prepared by mixing 0.3 mL of c3-Si (3), 2-Si (2) and 1-Si (1) in DMSO with 0.7 mL of water respectively. The three samples were then scanned using a 3T scanner. The T1 weighted axial images were obtained with a Fast Spin Echo sequence. Also, longitudinal relaxation times T_1_ of the three contrasts were determined using a 3T whole body Siemens scanner via inversion recovery techniques. These were then converted into longitudinal relaxation rates ($R_{1}=1/T_{1}$).

**Figure 3:**
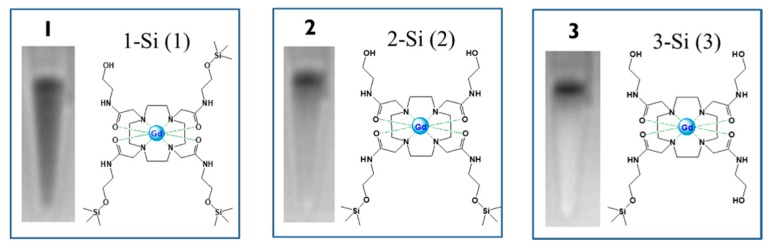
*T_1_* weighted images of (**1**) Gd(III)-DOTA-silyl complex with one cleaved silyl group; (**2**) Gd(III)-DOTA-silyl complex with two cleaved silyl group; (**3**) Gd(III)-DOTA-silyl complex with three cleaved silyl groups.

As is illustrated in both Figure 3 and Table 3, the minimum number of silyl groups that must be removed in order to obtain a readable signal is 3, which corroborates with the assumption data obtained above (Table 2).

**Figure 4:**
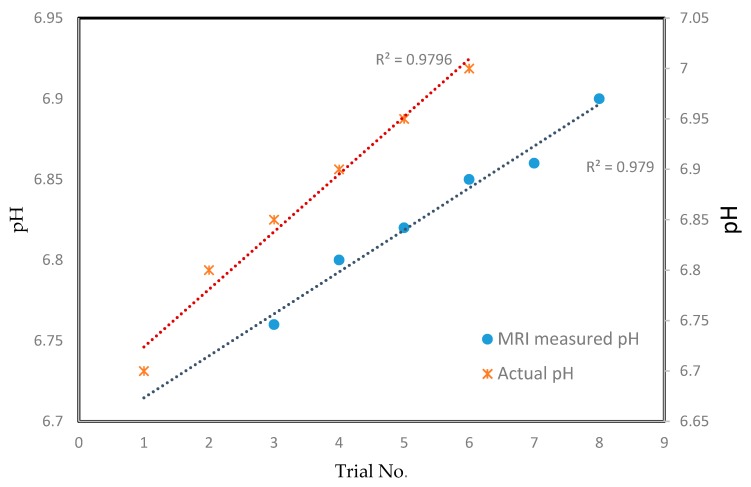
Linear relationship line for the actual pH vs. the magnetic resonance imaging (MRI) measured pH.

Also, it was revealed that excellent linearity was obtained by the developed technique within the tested pH range with an R-squared value of 0.979—that is, almost identical to the actual pH—as demonstrated in Figure 4. The results obtained therefore confirm that the developed method can precisely estimate pH.

**Figure 5:**
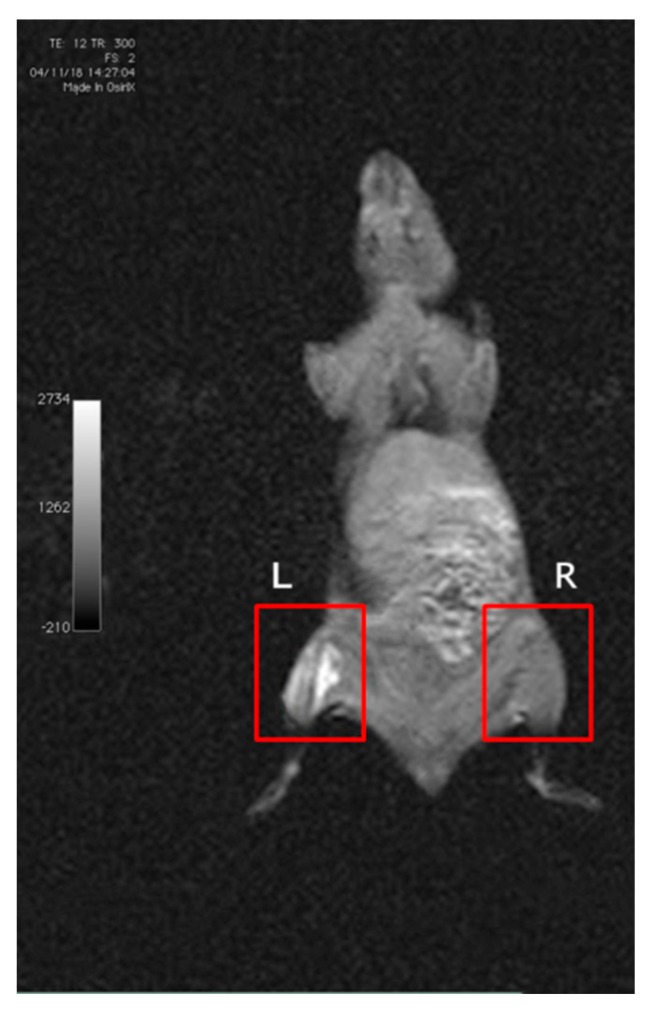
T1-weighted image acquired using scans of mice injected with Gd(III)-DOTA-Silyl contrast. L: left leg injected first with lactic acid buffer (pH 5.5) then with the pH sensitive contrast agent. R: right leg injected only with the pH sensitive contrast agent.

In order to examine the capability of this new contrast agent to respond to in vivo pH change, the left leg (L) of a healthy mouse was injected with a lactic acid buffer (pH 5.5), while the other leg was left as a control. Then a solution of Gd(III)-DOTA-Silyl contrast agent was injected directly (5 mmol/kg) in both legs of the mouse. $T_{1}$-weighted MR images were acquired shortly after these injections. As depicted in Figure 3, a stronger $T_{1}$ signal was observed from the left leg (Figure 5 L). To the contrary, no obvious effects were shown in the mouse’s right leg (Figure 5 R), due to the absence of lactic acid in the tissue. These observations undoubtedly demonstrate the ability of the contrast agent to sense the change in pH.

**Figure 6:**
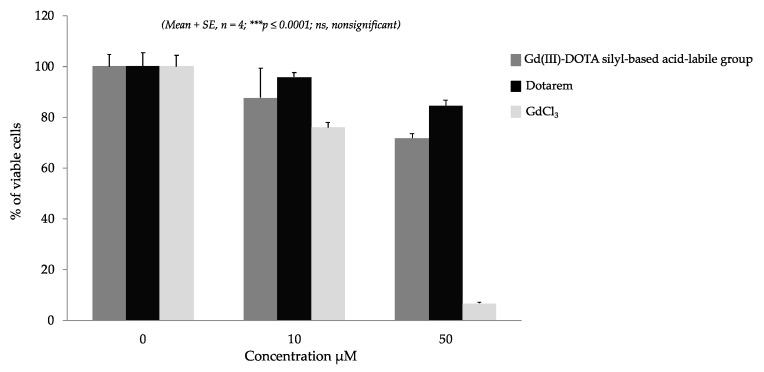
Cytotoxicity comparison of Gd(III)-DOTA-silyl-based acid-labile group, Dotarem (commercial MRI contrast) and GdCl_3_ free from at different concentrations (0, 10 and 50 μM) using MTT assay on Jurkat human leukemia cell line.

To compare the cytotoxicity of Gd(III)-DOTA-silyl-based acid-labile group, Dotarem (commercial MRI contrast) and GdCl_3_ free from, MTT assay is performed to evaluate the cells viability, on the Jurkat leukemia cell line with different concentrations of these three candidates. In doing so, we demonstrate that the toxicity of both contrasts Gd(III)-DOTA-silyl-based acid-labile group and Dotarem, which represent the contrast in both forms with and without the silyl groups, are greatly reduced compared to the GdCl_3_ free form at high concentrations (50 μM). The contrast between cytotoxicities was particularly obvious at 50 μM. In the case Gd(III)-DOTA-silyl-based acid-labile group, Dotarem the Jurkat leukemia cells remained viable after 3 days, whereas most cells treated with GdCl_3_ free from were dead (Figure 6). These results clearly confirm the biosafety of this agent.

**Figure 7:**
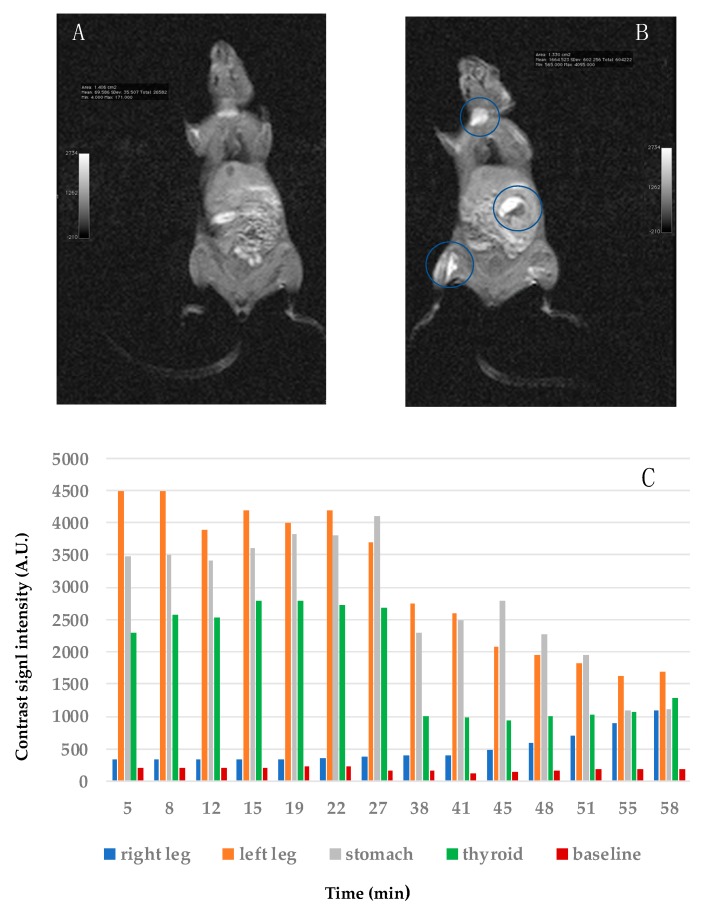
(**A**) Before contrast agent injection (**B**) T1-weighted image acquired using scans of mice injected with Gd(III)-DOTA-Silyl contrast after 90 min. (**C**) Diodistribution of Gd(III)-DOTA-Silyl contrast within the mouse model at various time laps.

A preliminary experiments of the biodistribution, was performed by using healthy female mice of 6–8 weeks of age which has been injected with a solution of Gd(III)-DOTA-Silyl contrast agent (10 mmol/kg) in both mouse legs muscles, it is worth pointing that the left legs was injected with saline acidic solution 5 min prior to contrast injection. After 30 min from the injection and for a period of almost an hour, $T_{1}$-weighted MR images were acquired. As illustrated in Figure 7A,B, we can clearly observe a stronger $T_{1}$ signal from the left leg due to the cleavage of the silyl groups. A similar observation was made, as more of these contrast agents reach other acidic tissues such stomach, small part of the intestine and kidneys, also we observed a strong accumulation of these contrasts in thyroid gland, where the signal gained more strength with time Figure 7C. However, a closer examination of the biodistribution and clearance profile are under through investigation with animal disease model to better understand the mechanistic involved in this process.

## 3. Materials and Methods

All chemicals were of the highest purity possible. All chemicals were used as received and no further purification was performed. 2-aminoethanol, Trimethylsilane chloride, 2- Chloroacetyle chloride, Cyclen, GdCl_3_ were purchased from Sigma-Aldrich. GraphPad Prism software was purchased from GraphPad Software, (San Diego, CA, USA).

### 3.1. Synthesis of Compound (1)

2-aminoethanol (1.22 g, 20.0 mmol) and trimethylamine (4.05 g, 40.0 mmol) were dissolved in 40 mL chloroform. Trimethylsilane chloride (3.26 g, 30.0 mmol) was then added dropwise over a 5 min period, and the reaction mixture was stirred for 12 h at room temperature. After completion, the reaction mixture was washed with water (2 × 20 mL), and the organic phase was collected and dried over Na_2_SO_4_. The crude mixture was then purified by column chromatography using DCM as an eluent to render the desired product 1 as 2.37g of pale-yellow liquid (90% yield). 1H-NMR (600 MHz, CDCl_3_), 0.1 (s, CH3), 2.67 (t, *J* = 5.4 Hz, CH2-NH2), 3.49 (t, *J* = 5.4 Hz CH2-O), *m/z* GC-MS (EI) for C_5_H_15_NOSi: 133. Found [M] ^+^ = 133.

### 3.2. Synthesis of Compound (2):

Chloroacetyle chloride (2.26 g, 30.0 mmol) was added dropwise to a solution of compound **1** (2.67 g, 20.0 mol) and trimethylamine (4.05 g, 40.0 mmol) in 40 mL chloroform and the reaction mixture was stirred for 2 h at room temperature. After completion, the reaction mixture was washed with water (2 × 40 mL) and the organic phase was dried over Na_2_SO_4_. The crude mixture was then purified by column chromatography using DCM as an eluent to render the desired product **2** as 3.14g of pale-yellow liquid (88% yield). 1H-NMR (600 MHz, CDCl_3_), 0.1 (s, Si-CH3), 3.4 (q, *J* = 5.4 Hz, CH2-NH), 3.65 (t, *J* = 5.4 Hz CH2-O), 4.02 (s, CH2-CO); *m/z* GC-MS (EI) calculated for C_7_H_16_ClNO_2_Si: 209, Found [M]^+^ = 209.

### 3.3. Synthesis of Compound (3):

Cyclen (344 mg, 2.0 mmol). DIPEA (1.04 g, 8 equivalents) and compound 2 (3.4 g, 8 equivalents) were dissolved in 40 mL acetonitrile and the reaction mixture was refluxed for 12 h. After completion, acetonitrile was removed under reduced pressure and the crude mixture was purified by column chromatography using DCM as an eluent to render the desired product **3** as 131mg of white solid (92% yield). 1H-NMR (600 MHz, CDCl3), 0.1 (s, Si-CH3), 3.05 (q, *J* = 7.2 Hz, 4xCH2-NH-dota), 3.37 (q, *J* = 5.4 Hz, 4 × CH2-NH), 3.65 (t, *J* = 5.4 Hz 4 × CH2-O), 4.0 (s, 4 × CH2-CO); *m/z* MALDI-TOF exact molecular weight 864. Found 887 [M + Na] ^+^.

### 3.4. Synthesis of Compound (4):

GdCl_3_ (53 mg, 0.2 mmol) was added to a solution of compound **3** (173 mg, 0.2 mmol) in 20 mL acetonitrile. The reaction mixture was refluxed for 12 h. After completion, acetonitrile was removed under reduced pressure to obtain Gd-DOTA complex **4** as 174mg of white solid (85% yield). Elemental analysis, exact percentage for C_36_H_80_GdN_8_O_8_Si_4_, C: 42.28; H: 7.88; Gd: 15.38; N: 10.96; O: 12.52; Si: 10.99. Found percentage: C: 42.88; H: 8.21; Gd: 15.07; N: 10.37; O: 12.03; Si: 10.49. *m/z* MALDI-TOF exact molecular weight 1022.7. Found [M + Na] ^+^ = 1045.

### 3.5. Testing the Ability of Gd(III)-DOTA-Silyl Contrast to Probe pH

The in vitro imaging effects of Gd(III)-DOTA-Silyl were visualized on a 3 Tesla whole body Siemens scanner (MAGNETOM Prisma, Siemens Healthcare) with $T_{1}$-weighted images using the Turbo Spin Echo (TSE) sequence. The experiment intended to assess the sensitivity of the new contrast Gd(III)-DOTA-Silyl to acid concentration change, and was performed using 8 tubes prepared from different lactic acid concentration with the following pH (6.5, 6.6, 6.7, 6.8, 6.85, 6.9, 6.95 and 7). The pH of the samples was determined using the following Equation (4):

(4) $$K_{a}=\left[H^{+}\right]\left[lactate\right]/\left[lactic\;acid\right]$$

where *K_a_* = 1.38 × 10^−4^. A pH with an accuracy of ±0.01 pH units was also used to confirm these values. The ninth tube, with well-known Gd^+3^ concentration (Gd(III)-DOTA, 7.6 × 10^−8^ mol/L), was used as reference sample.

### 3.6. Synthesis of Compounds (3-Si (**3**), 2-Si (**2**), 1-Si (**1**)):

Compound (**3**) (3 g, 9.78.10^−4^ moles) was dissolved in 30 mL DCM. The mixed solution was aliquoted into three separate flasks. Then, at a very slow rate (1 drop/min), 1 equivalent, 2 equivalents and 3 equivalents of acetic acidic solution (10 mL) were added to the three flasks respectively while stirring strongly. The resultant mixture was stirred for 1 h at room temperature. After completion, DCM was removed under reduced pressure and the crude mixture was purified by column chromatography using DCM/hexane as an eluent. The yield of these DOTA derivatives are low due to the lack of selectivity, nonetheless the aim of these reactions is not yield but having these derivatives to test our assumption concern the required number for an efficient shielding. For the first compound (1-Si (**1**’)), with 3 silyl protecting groups, yield was found to be 31%. For the second compound (1-Si (**2**’)), with 2 silyl protecting groups, the yield was 37%. For the third compound (1-Si (**3**’)) with the 1 silyl protecting group, the yield was 33%. No amide group hydrolysis was observed in the NMR spectrum during the silyl cleavage (data not shown).

1-Compound Si (**1**) 1H-NMR (600 MHz, CDCl3), 0.09 (s, 3 × Si-(CH3)_3_), 3.16 (q, *J* = 7.2 Hz, 8 × CH2-N-dota), 3.47 (q, *J* = 5.4 Hz, 4 × CH2-NH), 3.55 (t, *J* = 5.4 Hz, CH2-OH), 3.85 (t, *J* = 5.4 Hz 3 × CH2-O-Si), 4.2 (s, 4 × CH2-CO); *m/z* MALDI-TOF exact molecular weight 792. Found 831 [M +K]^+^.

1-Compound Si (**2**) 1H-NMR (600 MHz, CDCl3), 0.09 (s, 2 × Si-(CH3)_3_), 3.2 (q, *J* = 7.2 Hz, 8 × CH2-N-dota), 3.5 (q, *J* = 5.4 Hz, 4 × CH2-NH), 3.65 (t, *J* = 5.4 Hz, 2 × CH2-OH), 3.95 (t, *J* = 5.4 Hz 2 × CH2-O-Si), 4.3 (s, 4 × CH2-CO); *m/z* MALDI-TOF exact molecular weight 721. Found 760 [M +K]^+^.

1-Compound Si (**3**) 1H-NMR (600 MHz, CDCl3), 0.1 (s, Si-CH3), 3.15 (q, *J* = 7.2 Hz, 8 × CH2-N-dota), 3.46 (q, *J* = 5.4 Hz, 4 × CH2-NH), 3.55 (t, *J* = 5.4 Hz, 3 × CH2-OH), 3.85 (t, *J* = 5.4 Hz, CH2-O-Si), 4.35 (s, 4 × CH2-CO); *m/z* MALDI-TOF exact molecular weight 648.8 Found 671 [M + Na]^+^.

### 3.7. Complexation Reaction

GdCl_3_ (53 mg, 0.2 mmol) was added to each flask that contains respectively Compound Si (**1**), Compound Si (**2**) and Compound Si (**3**) that has been dissolved 20 mL dry acetonitrile. The reaction mixtures were refluxed for 12 h. After completion, acetonitrile was removed under reduced pressure to obtain the following complex Gd-DOTA complex with different number of protecting

Gd-DOTA Si (**1**) Elemental analysis, exact percentage for C_33_H_72_GdN_8_O_8_Si_3_, C: 41.70; H: 7.64; Gd: 16.45; N: 11.76; O: 13.42; Si: 8.89. Found percentage: C: 41.88; H: 7.21; Gd: 15.07; N: 11.57; O: 12.03; Si: 8.49. *m/z* MALDI-TOF exact molecular weight 950. Found [M + Na] ^+^ = 973.

Gd-DOTA Si (**2**) Elemental analysis, exact percentage for C_36_H_80_GdN_8_O_8_Si_2_, C: 41.70; H: 7.34; Gd: 17.90; N: 12.76; O: 14.57; Si: 6.40. Found percentage: C: 41.88; H: 7.21; Gd: 17.07; N: 12.57; O: 14.03; Si: 6.49. *m/z* MALDI-TOF exact molecular weight 878. Found [M + Na] ^+^ = 901.

Gd-DOTA Si (**3**) Elemental analysis, exact percentage for C_27_H_56_GdN_8_O_8_Si, C: 40.23; H: 7.00; Gd: 19.51; N: 13.90; O: 15.88; Si: 3.48. Found percentage: C: 40.57; H: 7.20; Gd: 19.01; N: 13.70; O: 15.18; Si: 3.18. *m/z* MALDI-TOF exact molecular weight 806. Found [M + Na] ^+^ = 829.

### 3.8. Gd(III)-DOTA-Silyl Contrast Agent *In Vitro* Assessment

The Gd(III)-DOTA-silyl capability to respond to any change in pH condition was assessed using two tubes with different pH (4 and 7). To these two tubes an equal amounts of Gd(III)-DOTA-silyl was added and the mixture was left to rest for 2 h MRI scanning. 3T scanner (MAGNETOM Prisma, Siemens Healthcare) with a single channel body coil for signal reception is used. A T1 weighted axial image was obtained with a Fast Spin Echo sequence with the following parameters: TR/TE = 250/2.46 ms; matrix size, 256 × 256 resolution and 8 slices with slice thickness 20 mm; FOV: 220 × 220 mm; flip angle: 70; receiver bandwidth set to 320 Hz/pixel and number of averages = 2.

### 3.9. T_1_ Weighted MR Images *In Vitro*

In vitro imaging of the Gd(III)-DOTA-3 silyl groups, Gd(III)-DOTA-2 silyl groups and Gd(III)-DOTA-2 silyl groups was obtained using a 3T scanner (MAGNETOM Prisma, Siemens Healthcare) with a single channel body coil for signal reception. A T1 weighted axial image was obtained with a Fast Spin Echo sequence with the following parameters: TR/TE = 250/2.46 ms; matrix size, 256 × 256 resolution and 8 slices with slice thickness 20 mm; FOV: 220 × 220 mm; flip angle: 70; receiver bandwidth set to 320 Hz/pixel and number of averages = 2.

### 3.10. Gd(III)-DOTA-Silyl Contrast Agent *In Vivo* Assessment

For animal experimentation European (Directive 86/609/EEC on the protection of animals used for scientific purposes) and the investigation protocols was approved by institution Ethics Committee (Approval Number: 241-2314). Mice were kept under constant temperature and fed ad lib autoclaved chow and water. The Gd(III)-DOTA-Silyl groups were also tested on a mouse. One mouse was injected (50 μL) in one hind left leg with 0.1 M lactic acid buffer (pH = 5.5). The other leg was not injected. Both mouse legs were then injected with an aqueous solution (water/DMSO, 60/40) of Gd(III)-DOTA-Silyl (5 mmol/kg). The mouse was positioned inside the MRI head coil and MR images were subsequently acquired.

### 3.11. MRI Protocol

In vitro assessment of Gd(III)-DOTA-Silyl were visualized on a 3 Tesla whole body Siemens scanner (MAGNETOM Prisma, Siemens Healthcare) with $T_{1}$ weighted images using Turbo Spin Echo (TSE) sequence [21,22]. Experiments were performed with a single channel body coil for signal reception. A $T_{1}$ weighted axial image was obtained with the TSE sequence using the following parameters: repetition time (TR)/echo time (TE) = 250/2.46 msec. The repetition time (TR) is necessary for the excited spins from the same slice to fully relax prior to the next excitation [21,22]. It represents the time between successive pulse sequences, and as given below it is strongly linked to the $T_{1}$ values of the excited spins. Spins with longer $T_{1}$ require longer TR values to relax and vice versa. The echo time (TE) is the time required for the excited spins to possess a phase coherence and give rise to a spin-echo signal. It determines the duration between the delivery of the Radio Frequency (RF) pulse and spin echo signal reception. It is, therefore, governed by the $T_{2}$ parameter of the excited spins. Pixel size of the image was set to 256 × 256 and 8 slices with slice thickness of 20 mm were imaged. The field of view (FOV) of the scanned region around the tubes was 220 × 220 mm; the flip angle of the excitation RF pulse was 70°; and the receiver bandwidth was set to 320 Hz/pixel.

### 3.12. MTT Assay to Assess Gd(III)-DOTA-Silyl-Based Acid-Labile Group Cytoxicity

To test Gd(III)-DOTA-silyl-based acid-labile group cytoxicity and compare it with the toxicity Gd(III)-DOTA (commercial MRI contrast) and GdCl3 free from, a Jurkat cells were inoculated in 96-well culture plate (100 μL in each well with a concentration of 2 × 105 cells/mL). Different concentrations of Gd(III)-DOTA-silyl-based acid-labile group, Gd(III)-DOTA (commercial MRI contrast) and GdCl3 free from, diluted in 50 μL of RPMI1640/ethanol (8:2) were added to the well. Each experiment was carried out in in quadruplicate. Then the cells were incubated for 3 days. The cytotoxicity of Gd(III)-DOTA-silyl-based acid-labile group, Gd(III)-DOTA (commercial MRI contrast) and GdCl3 free from was assessed by the MTT assay in accordance with the procedure mentioned in literature. The results were analyzed using GraphPad Prism software (Mean + SE, n = 4; *** *p* ≤ 0.0001; ns, nonsignificant).

## 4. Conclusions

In summary, this investigation demonstrated the ability of the newly developed pH sensitive contrast agents (Gd(III)-DOTA-Silyl) to probe endogenous pH using a single injection. The results obtained clearly show the feasibility of such a method to sense the pH of any given sample in a straightforward way and with high accuracy using MRI as a detection method. For the next stage of research, a thorough in vivo assessment study will be carried out using a mice tumor model instead of a lactic acid solution.

## Figures and Tables

**Table 1:** Calculated Gd concentration using $T_{1}$ weighted image intensity.

Tube	N1	N2	N3	N4	N5	N6	N7	N8
Amp (a.u)	0.85	0.83	0.79	0.76	0.68	0.67	0.55	0.50
[Gd^+3^].10^−8^ M	8.89	6.95	5.70	5.60	5.00	4.80	4.60	4.45

**Table 2:** pH calculated based on the assumption of 1, 2, 3 and 4 protons required to lift the shielding on the Gd. The variable pH (actual pH) represents the values measured by the pH meter. The *t*-value was calculated using the experimental pH values compared to the actual pH values.

Tubes	N1	N2	N3	N4	N5	N6	N7	N8	*t*-Value
pH (1 proton)	7.05	7.16	7.24	7.25	7.30	7.32	7.33	7.35	45.72
pH (2 protons)	6.74	6.85	6.94	6.96	7.00	7.01	7.03	7.05	15.42
pH (3 protons)	6.57	6.60	6.76	6.80	6.82	6.85	6.86	6.95	1.54
pH (actual pH)	6.50	6.60	6.70	6.80	6.85	6.90	6.95	7.00	NA

**Table 3:** Longitudinal relaxation time and relaxation rate of of 1) Gd(III)-DOTA-silyl complex with one cleaved silyl group; 2) Gd(III)-DOTA-silyl complex with two cleaved silyl groups; 3) Gd(III)-DOTA-silyl complex with three cleaved silyl groups.

CA	T_1_(ms)	R_1_×10^−3^(ms^−1^)
**3-Si (3)**	141.4	7.07
**2-Si (2)**	298.7	3.3
**1-Si (1)**	364.8	2.74
